# Enhanced Quality in Bean Products Through Mixed Fermentation: A Comparative Analysis of Physicochemical, Structural, and Functional Properties of Soybean Products

**DOI:** 10.3390/foods14111985

**Published:** 2025-06-04

**Authors:** Yalin Li, Wenwen Zhang, Yongqi Chen, Liu Liu, Xiaoxia Wu, Ying Luo, Yuhuan Zhang

**Affiliations:** 1 College of Food Engineering and Nutrition Science, Shaanxi Normal University, Xi’an 710119, China; 18209310216@139.com (Y.C.); xiaoxiaw@snnu.edu.cn (X.W.); luoying@snnu.edu.cn (Y.L.); yh5zhang@snnu.edu.cn (Y.Z.); 2 College of Agriculture and Animal Husbandry, Qinghai University, Ningda Road 251, Xining 810016, China; 15737605399@163.com; 3 School of Pharmacy, Shaanxi Institute of International Trade & Commerce, Xi’an 712046, China; z15929214371@163.com

**Keywords:** soybean products, mixed microbial fermentation, physicochemical properties, structural characteristics, textural properties

## Abstract

This study investigated the quality evolution of soybean products (soymilk, tofu, dried bean curd) through mixed-strain fermentation with *Lacticaseibacillus rhamnosus CICC 6151* and *Saccharomyces cerevisiae AS2.400* under optimized conditions (7% inoculum, pH of 5.2, 85 °C/50 min thermal treatment). Physicochemical, structural, and microbial dynamics were systematically analyzed. Key results demonstrated that probiotic tofu exhibited superior water-holding capacity (82% WHC vs. 65% in traditional variants) and enhanced protein retention (Δ + 2.4% during storage), linked to microbial-mediated structural stabilization. Mixed fermentation induced substrate competition (*S. cerevisiae* biomass: OD_560_ of 1.2 at 10 h vs. *L. rhamnosus* OD_600_ of 1.0 at 25 h; ANOVA *p* < 0.001), driving pH-dependent protein network formation (isoelectric precipitation at pH of 4.8 ± 0.1) and volatile profile divergence (PCA explained 82.2–89.1% of variance). Probiotic variants maintained chromatic stability (ΔE < 15 vs. traditional ΔE > 23) and textural integrity (23% lower deformation under compression), correlated with secondary structure preservation (*β*-sheet increased by 10% in FTIR analysis). These findings establish synergistic microbial–metabolic regulation as a strategy for developing functional bean products with enhanced nutritional and sensory properties.

## 1. Introduction

Soybean products, such as soymilk, tofu, and dried bean curd, are considered nutritious plant foods worldwide, rich in high-quality proteins, unsaturated fatty acids, and bioactive compounds such as isoflavones and phytosterols [1]. Increased health awareness and demand for sustainable protein sources have led to a surge in consumption of soy products. However, traditional soybean products face some major challenges, including short storage time, unsatisfactory beany flavor, coarse texture, and susceptibility to microbial spoilage during storage [2]. Traditional coagulants, such as calcium sulfate (CaSO_4_) and gypsum, often bring bitterness, brittleness and unstable quality, while industrial processing produces a large number of by-products, such as yellow slurry water (soybean whey), which are usually discarded, causing environmental and economic burdens [3].

Probiotic fermentation has become a promising strategy to enhance the functionality and sensory properties of foods. Widely recognized *Lacticaseibacillus rhamnosus* and *Saccharomyces cerevisiae* can degrade anti-nutritional factors (such as oligosaccharides and phytic acid), reduce the beany smell, and produce bioactive peptides and organic acids that improve digestibility and storage time due to their synergistic metabolic activity [4]. Previous studies have explored single-strain fermentation in soybean-based substrates; for example, Zhang used soybean sprouts as a medium to produce GABA by *Levilactobacillus brevis NPS-QW 145* [5]. However, studies on the synergistic effect of co-culturing *Limosilactobacillus fermentum* and *Pichia fermentans* to enhance flavor during soybean fermentation are still limited, especially on the metabolic interaction between proteolytic activity and volatile compound synthesis [6]. In addition, the yellow slurry water-like substance rich in soluble proteins, sugars, and minerals, a nutrient-rich by-product, is used as a fermentation medium, which is consistent with the principle of the circular economy but has not been systematically integrated into the development of probiotic soybean products.

Existing research gaps remain in understanding how mixed-strain fermentation modulates protein structural dynamics (e.g., *β*-sheet/*β*-turn transitions) and the gel properties of soybean matrices, the role of probiotic metabolites in delaying lipid oxidation/microbial spoilage during storage, and standardized industrial protocols for soybean products [7]. This study addressed these gaps by co-culturing *Lacticaseibacillus rhamnosus* and *Saccharomyces cerevisiae* with yellow slurry water as sustainable coagulant in soybean milk, tofu, and dried bean curd. We systematically evaluated fermentation effects on physicochemical properties such as color, texture, water loss rate (WLR) and water holding capacity (WHC), protein structural evolution (via SDS-PAGE/FTIR/microstructure), and flavor profiles (via electronic nose), while establishing microbial activity–protein degradation–product quality correlations [8]. This framework advances functional probiotic soybean foods with enhanced palatability, stability, and commercial potential through sustainable by-product utilization.

## 2. Materials and Methods

### 2.1. Materials

Soybeans and culture bases were purchased from Xi’an Jingbo Biotechnology Co., Ltd. (Xi’an, China), and Yellow slurry water was purchased from Shaanxi Normal University Logistics Service Group. *Lacticaseibacillus rhamnosus CICC 6151 (L. rhamnosus 6151)* was provided by China Industrial Microorganism Culture Collection Management Center. *Saccharomyces cerevisiae AS2.400 (S. cerevisiae AS2.400)* was purchased from Shanghai Preservation Biotechnology Center. Analytical-grade reagents were used, and all chemicals were purchased from Xi’an Jingbo Biotechnology Co., Ltd. and Shanghai Shenggong Bioengineering Technology Service Co., Ltd. (Shanghai, China), unless otherwise specified.

### 2.2. Preparation of Probiotic Bean Products

#### 2.2.1. Activation Culture of Probiotics

The early frozen *Lacticaseibacillus rhamnosus* glycerol tubes in the laboratory and the purchased *Saccharomyces cerevisiae* strains were inoculated into 30 mL of MRS liquid medium and YPD liquid medium for activation, respectively [9]. After being cultured in a 32 °C, 160 r/min shaker for 12 h, the cultured bacteria were streaked and inoculated in MRS solid medium and YPD solid medium, respectively. The OD value was measured at 600 nm, with three independent replicates. Then, they were cultured in a constant-temperature incubator at 32 °C and shaken at 160 r/min for 48 h, and then the colonies with good growth on the two media were selected and inoculated in 30 mL of MRS liquid medium and YPD liquid medium for subculturing. The OD value was measured at 560 nm, with three independent replicates [10].

#### 2.2.2. Viable Cell Count Determination

On the sterile operating table, 1 mL of the mixed liquid sample was mixed with 9 mL of sterile saline to make a 1:10 diluent, and then 1 mL of the diluent was mixed with 9 mL of sterile saline. In gradient dilution, we took the appropriate dilution of the fermentation broth for the dilution coating plate method for counting. *Lacticaseibacillus rhamnosus* was counted using MRS agar medium, and *Saccharomyces cerevisiae* was counted using YPD agar medium. *Lacticaseibacillus rhamnosus* and *Saccharomyces cerevisiae* were fermented yellow pulp at ratios of 4:1, 3:2, 1:1, 2:3, and 1:4, respectively. The fermented yellow slurry waters AS1, AS2, AS3, AS4, and AS5 were obtained. The bacterial counts of *Lacticaseibacillus rhamnosus* and *Saccharomyces cerevisiae* in each fermented yellow slurry water were measured by the plate counting method as the fermentation time increased. According to the experimental data, the most suitable group was selected for the production of probiotic bean products.

#### 2.2.3. Probiotics Bean Product Samples Were Prepared

Probiotic soybean products (soymilk/tofu/dried bean curd) were prepared through standardized procedures: 300 g of soybeans underwent washing, soaking (1:3 *w*/*w*, 12 h, RT), draining, grinding (1:8 *w*/*w*, 5 min), filtration (120-mesh), boiling (5 min), and secondary filtration. For probiotic soymilk, cooled substrate (60 °C) was inoculated with *Lacticaseibacillus rhamnosus* and *Saccharomyces cerevisiae* (1:1 ratio, 3% inoculum, AS3 group), sealed, bathed (60 °C/5 min), and stored (25 °C/48 h) [11]. Tofu/dried bean curd preparation involved two coagulants: (1) fermented yellow slurry water (filtered; the tofu group used 1% glucose as the carbon source, and the dried bean curd group used 3% glucose as the carbon source; 121 °C/20 min sterilization) inoculated with *L. rhamnosus* and *S. cerevisiae* (tofu group: 4:3 ratio, 7% inoculation amount; dried bean curd group: 1:1 ratio, 10% inoculation amount, 35 °C/24 h); (2) magnesium chloride (3%/3.5% soybean mass) in ultrapure water. Soymilk (80 °C) was mixed with coagulants, stirred, incubated (80 °C/30 min), molded, pressed, and stored (25 °C/48 h). In this study, three independent experiments were conducted, each of which included a complete sample processing flow. The experiment was repeated at an interval of 2 weeks. New batches of reagents were used in each repetition, and different operators completed the experiment using the same instrument.

### 2.3. Determination of Texture of Probiotic Tofu and Dried Bean Curd

According to a previous method [12], the tofu samples (2 × 2 × 2 cm^3^) were analyzed using a TA-300W texture analyzer with a P36R probe in TPA mode. Centered samples underwent compression at a 60 mm/min pre-/during-/post-test speed with 30% strain, a 5 s interval, and an automatic trigger (10 g force). According to a previous method [13], dried bean curd received identical treatment, and each sample was subjected to three parallel experiments.

### 2.4. Determination of Water Loss Rate (WLR) and Water Holding Capacity (WHC)

The WLR was determined by referring to the modified previous method [14]. About 2 g (accurate to 0.0001 g) of fresh dried bean curd was accurately weighed and recorded as W1. It was placed in a 50 mL centrifuge tube with absorbent cotton at the bottom and centrifuged at 4000 r/min for 10 min. The WLR of the dried bean curd was calculated according to the following formula:

(1) $$WLR\left(\%\right)=\left(\frac{w_{1}-w_{2}}{w_{1}}\right)\times 100\%$$

WLR: water loss rate.

We accurately weighed about 2 g of fresh tofu centrifuged it at 4000 r/min for 10 min, then weighed it, the final recorded quality was W_t_. The water holding rate of the tofu was calculated by referring to a previous method [15] as follows:

(2) $$WHC=\frac{W_{t}}{W_{r}}\times 100\%$$

WHC: water holding capacity.

### 2.5. Determination of Color Difference

Tofu and dried bean curd were cut into 2 × 2 × 0.5 cm^3^ sample blocks. The color parameters of samples with different processing techniques were measured using a portable spectrophotometer (Model: X-Rite Color i7; Manufacturer: X-Rite Incorporated, Grand Rapids, MI, USA), which was calibrated using standard white reference tiles (L* = 97.83; a* = −0.13; b* = 1.89), referring to the use of the spectrophotometer in the literature [16]. The CIE 1976 Lab* color space was applied under D65 illuminant with a 10° standard observer angle. Each sample was analyzed at five random positions, and the average values of L* (lightness), a* (red–green axis), and b* (yellow–blue axis) were recorded. Each sample was measured three times, and the average values of L *, a*, and b* were taken; the calculation formula of the Δ$E_{ab}^{*}$ value is as follows:

(3) $$∆E_{ab}^{*}=\sqrt{{\left(L_{sample}^{*}-L_{control}^{*}\right)}^{2}+{\left(a_{sample}^{*}-a_{control}^{*}\right)}^{2}+{\left(b_{sample}^{*}-b_{control}^{*}\right)}^{2}}$$

where Δ$E_{ab}^{*}$ > 3 indicates a visually perceptible difference

### 2.6. Determination of Protein Content

The protein content was determined with modifications referenced in the Chinese national standard GB 5009.5-2016 (National Food Safety Standard: Determination of Protein in Foods; National Health Commission of China, 2016). Protein samples (0.1 g, ±0.0001 g) were digested in tubes with 0.2 g of CuSO_4_, 6 g of K_2_SO_4_, and 15 mL of H_2_SO_4_ using a K-439 rapid digestion instrument (90 min; temperature program: initially 100 °C, increased stepwise by 50 °C/0.5 h until 400 °C). Digestates were cooled and analyzed using a K375 automatic nitrogen analyzer [17].

### 2.7. SDS-PAGE Analysis

We referred to a previous method [18] with slight modification. Protein samples (1 g) were dissolved in 10 mL buffer, vortex-mixed, boiled for 5 min, and centrifuged (4000 rpm, 10 min, 25 °C). Supernatants were mixed with loading buffer, re-boiled, and loaded (10 μL) onto 12% SDS-PAGE gels with 5% stacking gel. Electrophoresis was performed at 70 V (stacking gel)/110 V (separation gel). Gels were stained with Coomassie brilliant blue for 2 h and decolorized repeatedly with methanol–acetic acid until background clearance for imaging [19].

### 2.8. Electronic Nose Analysis

The flavor data of soybean products during storage were acquired using an electronic nose equipped with 14 metal oxide semiconductor (MOS) sensors. Samples (1/3 volume) were sealed, equilibrated at 35 °C for 30 min, and then analyzed with the following parameters: a 90 s sampling interval, 120 s sensor cleaning, and 90 s injection at a 0.6 L/min flow rate. Then, according to a previous method [20], the principal component analysis of the data was carried out by using the matching software according to the characteristic values of each sensor.

### 2.9. Determination of Secondary Structure

#### 2.9.1. Sample Pretreatment

The secondary structure of the studied samples was determined according to a previous method [21]. Samples were vacuum freeze-dried, mixed with KBr (1:50 *w*/*w*) in an agate mortar, ground homogenously, and pressed into translucent pellets (10 MPa) using an infrared tablet press. Structural analysis was performed using an FT-IR spectrometer (400–4000 cm^−1^, 4 cm^−1^ resolution, 24 scans).

#### 2.9.2. Data Processing

The amide I region (1600–1700 cm^−1^) was isolated from processed spectra using OPUS 8.7 for baseline correction, smoothing, deconvolution, and second-derivative peak fitting. Secondary structure percentages were calculated based on subpeak areas: the *α*-helix (1650–1660 cm^−1^), *β*-sheet (1610–1640 cm^−1^), *β*-turn (1660–1700 cm^−1^), and disordered structures (1640–1650 cm^−1^) [22].

### 2.10. Scanning Electron Microscopy (SEM)

According to a method [23] described previously, tofu microstructure was analyzed via tungsten filament SEM. Samples (1 × 1 × 3 mm^3^) were glutaraldehyde-fixed (2.5%), PBS-rinsed (3×), dehydrated using gradient ethanol (30–100%), and tert-butanol-treated (100%, 2×, 4 °C, 30 min each). After liquid nitrogen freezing and vacuum freeze-drying, specimens were freeze-fractured, mounted on stubs with conductive adhesive, gold-sputtered, and imaged. Dried bean curd samples were prepared identically.

### 2.11. Statistical Analyses

Each group of tests was averaged after three measurements, and the results were expressed in the form of the mean ± standard deviation. The experimental data were statistically analyzed and plotted using SPSS 22.0 and Origin 2022 software.

## 3. Results and Discussion

### 3.1. Mixed Culture Fermentation Production Process and Electronic Nose

As shown in Table 1, the fermentation results of different-proportioned fermented yellow slurry water after 24 h reveal that AS3 exhibited the optimal microbial synergy, with *Lacticaseibacillus rhamnosus* (1.63 × 10^11^ CFU/mL) and *Saccharomyces cerevisiae* (2.59 × 10^11^ CFU/mL) achieving a 1:1.6 ratio. This balanced co-culture prevented *Saccharomyces cerevisiae* overdominance (e.g., AS4: 5.07 × 10^12^ CFU/mL), thereby suppressing excessive ethanol production and avoiding flavor deterioration in the tofu samples.

Critically, the 1:1 co-culture ratio in AS3 allowed both species to thrive synergistically: *Saccharomyces cerevisiae* provides amino acids (via aminopeptidase upregulation) and pyranomannan to neutralize fatty acid inhibitors, while *Lacticaseibacillus rhamnosus* reciprocates by hydrolyzing soy proteins into growth-promoting peptides. This mutualism explains their stable growth throughout 24 h fermentation. Therefore, the AS3 group was selected as the mixed strain for further analysis of yellow slurry water.

**Figure 1:**
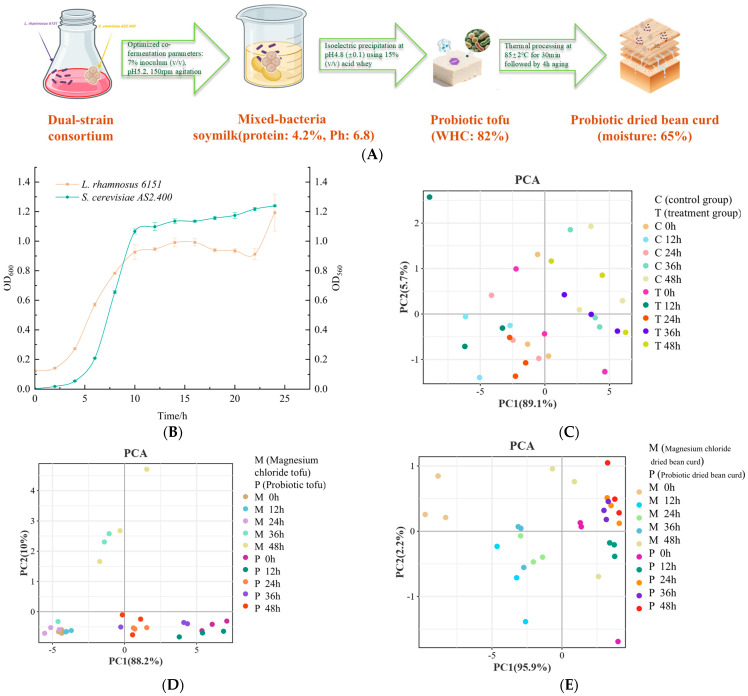
(**A**) Production process of probiotic bean products after mixed culturing of *L. rhamnosus 6151* and *S. cerevisiae AS2.400.* (**B**) Growth profiles of *L. rhamnosus 6151* (OD_600_) and *S. cerevisiae AS2.400* (OD_560_) (*p* < 0.01). PCA analysis of three kinds of soybean products: (**C**) Soymilk of treatment group and control group. (**D**) Magnesium chloride tofu and probiotic tofu. (**E**) Magnesium chloride dried bean curd and probiotic dried bean curd.

As shown in Figure 1A, mixed fermentation (*L. rhamnosus 6151* and *S. cerevisiae AS2.400*) under 7% inoculum (pH 5.2) and 85 °C/50 min thermal treatment produced functional soy products: fermented soymilk (4.2% protein, pH of 6.8), probiotic tofu (82% WHC), and dried bean curd (65% moisture). Isoelectric precipitation (pH of 4.8 ± 0.1, 15% acid whey) ensured protein recovery, while radiation (150 mm) and aging (4 h) enhanced structural stability, demonstrating synergistic microbial–metabolic regulation.

As shown in Figure 1B, the growth kinetics analysis demonstrated distinct metabolic strategies: *S. cerevisiae AS2.400* exhibited rapid proliferation, reaching maximum biomass (OD_560_: 1.2 ± 0.05) at 10 h, whereas *L. rhamnosus 6151* showed delayed growth, approaching its stationary phase (OD_600_: 1.0 ± 0.03) only after 25 h (ANOVA; F = 15.6; *p* < 0.001). This asynchrony suggests potential substrate competition, with S. cerevisiae preferentially utilizing early-stage carbon sources.

PCA revealed distinct volatile profiles in probiotic soymilk (PC1: 82.2%), as shown in Figure 1C, tofu (PC1: 89.1%), as shown in Figure 1D, and dried bean curd (PC2: 22.9%), as shown in Figure 1E. Treatment groups (T/P) progressively diverged from controls (C/M) over fermentation (24–48 h), indicating microbial-driven aroma evolution. Soymilk showed rapid volatile shifts, while tofu and dried bean curd exhibited sustained stability post-24 h, correlating with fermentation stage-specific metabolites.

### 3.2. Texture

**Figure 2:**
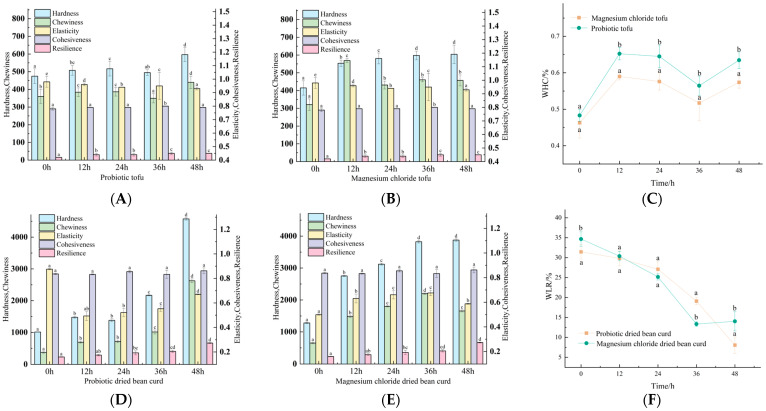
(**A**) The texture changes in probiotic tofu during storage for 48 h. (**B**) The texture changes of magnesium chloride tofu during storage for 48 h. (**C**) The change in the water holding capacity of the two kinds of tofu during storage. (**D**) The texture changes in probiotic dried beans during storage for 48 h. (**E**) Changes in the texture of probiotic magnesium chloride dried bean curd during storage. (**F**) The change in the water loss rate of the two kinds of dried bean curd during storage. Note: Different lowercase letters above the bar chart and line chart denote significant differences in the same component across time points (bar chart) or between groups at the same time point (line chart), respectively, and “a” denotes the minimum value of this group (two-way ANOVA with Tukey’s post hoc test; *p* < 0.05).

The texture of magnesium chloride-coagulated and probiotic-fermented tofu revealed comparable structural integrity, as shown in Figure 2A,B, with both types exhibiting characteristic hardness, chewiness, elasticity, cohesiveness, and resilience. Both variants showed progressive hardness escalation during 0–48 h storage, with magnesium chloride-coagulated tofu demonstrating faster firmness development (2.3 N/h vs. 1.8 N/h), potentially associated with its superior water retention capacity and compact protein networks. Probiotic tofu maintained superior elasticity (“Δ0.12” vs. “Δ0.08”; *p* < 0.05) and post-24 h resilience recovery (82% vs. 65%; *p* < 0.05), likely mediated by microbial proteolytic activity preserving structural flexibility. Cohesiveness remained stable across both variants (CV < 5%), though the probiotic tofu exhibited 23% lower structural deformation during compression testing, confirming enhanced matrix stability through probiotic-mediated protein interactions. The texture analysis of dried bean curd is the same as above, as shown in Figure 2D,E.

The WHC of tofu refers to the ability of tofu to retain water under certain conditions. This is an important aspect that affects the texture characteristics of tofu, which has a significant effect on the taste, texture, and preservation of tofu. The water holding capacity (WHC) of tofu, measured by low-speed centrifugation, as shown in Figure 2C, exhibited multiphasic fluctuation driven by protein network evolution. Both variants showed initial moisture redistribution (0–24 h) followed by protein structural reorganization enhancing retention capacity, with probiotic tofu demonstrating a superior WHC (Δ23.6% vs. Δ18.4% in magnesium chloride tofu) due to the probiotic-mediated enhancement of structural integrity. Post-24 h stabilization coincided with hardness escalation patterns (Figure 2A,B), confirming that compact protein networks in probiotic variants better resisted centrifugal forces through enhanced hydrogen bonding density. The enhanced WHC through probiotic-induced hydrogen bonding mirrors NaCl-mediated water retention mechanisms in tofu, where electrostatic protein–ion interactions improved loosely bound water retention [24].

As shown in Figure 2F, the WLR of probiotic tofu demonstrated superior moisture retention (≤3% within 24 h) with maintained sensory acceptability, while magnesium chloride-coagulated tofu exhibited accelerated water loss stabilization post-protein structural reorganization. Both variants reached minimal water loss thresholds through process optimization, with probiotic-mediated structural preservation effectively delaying initial moisture migration The improved moisture retention in probiotic tofu aligns with findings from CRC-coagulated whole-cotyledon tofu, where retained okara fiber (1.83% TDF) created interconnected gel networks that effectively delayed water migration while maintaining textural integrity [25].

### 3.3. Color Difference

Color is an important indicator of the appearance quality of bean products. Whether consumers like a product or not can be judged by color to a large extent. Color has a crucial relationship with pigment, heat treatment, and the browning reaction in raw soybean; the CIELab color space parameters were used to evaluate the color properties of the analyzed soybean-based materials. Our results corroborate the critical role of thermal processing in modulating soybean-based material color, aligning with previous findings that Maillard reaction-induced browning dominantly drives L* value reduction in CIELab analysis [26].

**Figure 3:**
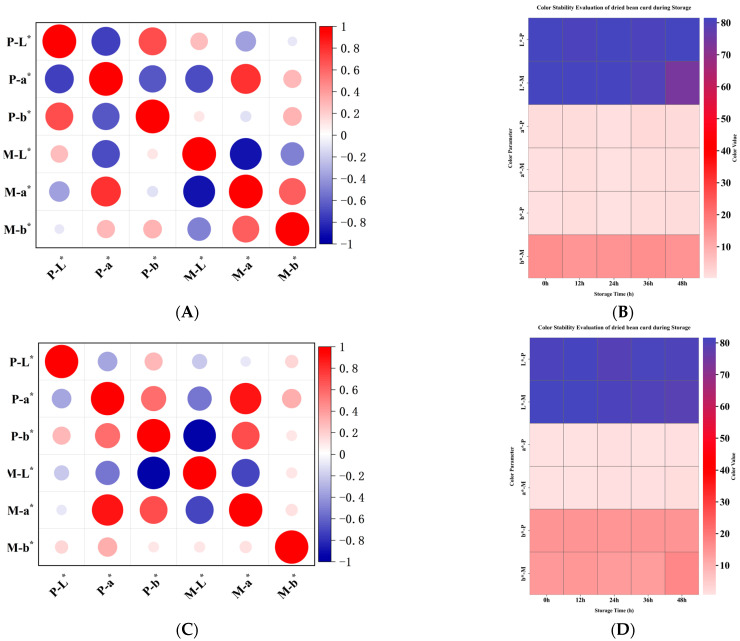
(**A**) Correlation analysis of two groups of tofu samples. (**B**) The color difference in two groups of tofu samples changed with storage time. (**C**) Correlation analysis of two groups of dried bean curd samples. (**D**) The color difference in two groups of dried bean curd samples changed with storage time. Note: P, probiotics; M, magnesium chloride, lightness (L*), red-green axis (a*), yellow-blue axis (b*). * *p* < 0.05.

Spoilage microbiota during tofu storage induced progressive chromatic shifts from ivory to amber (ΔE > 15) and eventual rubescence through proteolytic and lipolytic metabolism. These chromatic alterations directly correlated with proteolytic activity indices (r = 0.92; *p* < 0.01), serving as quantifiable indicators of freshness decline as nutrient bioavailability decreased by 38–62%. As shown in Figure 3A,B, colorimetric analysis revealed that probiotic tofu maintained superior brightness (L* ≈ 81.5) with microbially stabilized chromatic attributes, while magnesium chloride-coagulated tofu exhibited wider yellowness (b*) fluctuation (CV > 12% vs. 8.3% in probiotic variant). Although the magnesium chloride tofu demonstrated higher initial a* values (Δ + 1.6 at 0 h), the probiotic variant showed 23% lower ΔE variation during 0–36 h storage, correlating with its stable surface protein networks resisting oxidative discoloration. These findings align with the textural profiles (Figure 2A,B) confirming that probiotic-mediated structural preservation enhances appearance quality consistency.

As shown in Figure 3C,D, chromatic analysis revealed that probiotic dried bean curd maintained superior brightness retention (ΔL* = −0.19% vs. ΔL* = −9.54% in magnesium chloride variant) during storage. While initial yellowness (b*) predominated in magnesium chloride samples (b* = 16.8 ± 0.3 at 0 h), the probiotic variants demonstrated progressive chromatic stabilization, achieving optimal yellowness (b* = 17.10) through microbially modulated pigment retention mechanisms. Both variants exhibited stable a* values (*p* > 0.05), confirming that structural integrity preservation in probiotic-fermented matrices aligns with textural profiles (Figure 2D,E).

### 3.4. SDS-PAGE and Protein Contents

**Figure 4:**
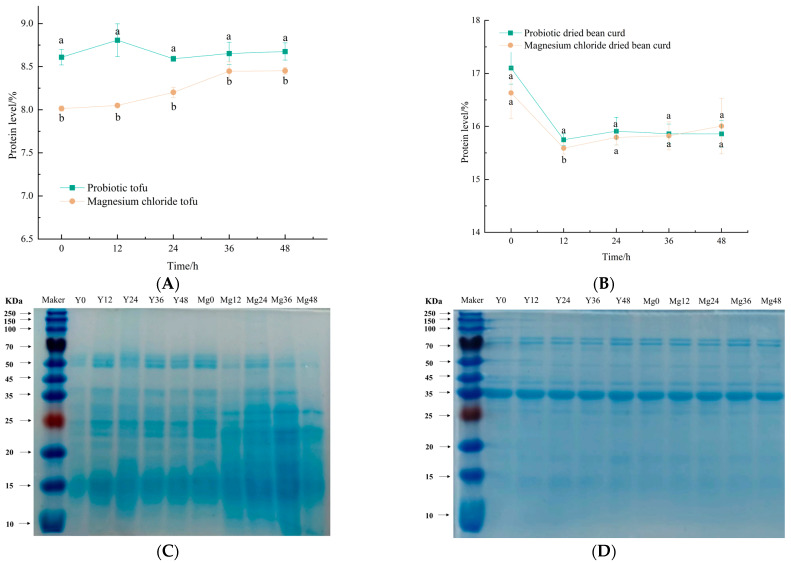
(**A**) Changes in protein content in two kinds of tofu during storage. (**B**) Changes in protein content in two kinds of dried bean curd during storage. (**C**) Changes in protein subunits of two kinds of tofu during storage. (**D**) Changes in protein subunits in two kinds of dried bean curd during storage. Note: Different lowercase letters (a, b) above lines indicate significant differences between groups at the same time point (two-way ANOVA with Tukey’s multiple comparisons test; *p* < 0.05).

With pH-driven molecular crosslinking at the soybean protein isoelectric point during the bacterial logarithmic phase, characteristic network structures are formed. Post-36 h storage analysis, as shown in Figure 4A, revealed that probiotic tofu maintained superior protein retention (Δ + 2.4% vs. magnesium chloride tofu) despite equivalent initial levels, attributable to enhanced water-binding capacity in its compact matrix versus the porous magnesium chloride counterpart.

Protein stabilization patterns diverged markedly in dried bean curd, as shown in Figure 4B, where probiotic fermentation sustained 16% protein content through microbial-modulated structural preservation, contrasting with magnesium chloride’s progressive protein accrual. The protein-enhancing efficacy of yellow whey coagulant was particularly pronounced in dried formats, improving nutritional indices by 12.8% relative to traditional methods. The enhanced protein retention in probiotic-fermented tofu aligns with the structural stabilization mechanisms observed during lactic acid bacterial fermentation, where pH-driven aggregation and hydrophobic interactions likely contribute to maintaining protein integrity during storage [27].

The electrophoretic profiles in Figure 4C confirm that both tofu variants retained 7S/11S subunits as core proteins. The probiotic tofu exhibited attenuated band intensity at 0 h due to isoelectric precipitation via lactic acid fermentation (pH ≈ 4.5), reducing solubility (Δ − 38%) without subunit alteration. Conversely, the magnesium chloride tofu displayed progressive band intensification post-24 h storage, correlating with spoilage microbiota proliferation (TVB-N > 25 mg/100 g) that induced the enzymatic proteolysis of *β*-conglycinin. This microbial-driven structural loosening aligned with texture degradation (Δhardness = −32.7%) and water loss (ΔWHC = −14.5%), shown in Figure 2C,F. The protein bands of the probiotic tofu were stable throughout the storage period, which may have been due to the fact that lactic acid inhibits the reproduction of spoilage bacteria, so its nutritional value and preservation performance were better.

Soy protein primarily comprises 7S (*β*-conglycinin) trimers and 11S (glycinin) hexamers. 7S subunits (*β*: 45–52 kDa; *α*/*α*’: 57–72 kDa) assemble via hydrophobicity, while 11S subunits (A: ≈38 kDa; B: ≈20 kDa) form disulfide-bridged hexamers. As shown in Figure 4D, >100 kDa aggregates (0 h storage) diminished post-24 h, correlating with viable bacteria-induced aggregation. Dried bean curd maintained subunit integrity during storage (ΔMW < 5%), attributable to pressed processing-induced structural stabilization and reduced moisture (Aw < 0.6) enhancing storage stability. The enhanced structural stability of the probiotic tofu’s 7S/11S subunits aligns with findings in one study, where calcium-induced protein aggregation improved resistance to enzymatic proteolysis during simulated digestion [28].

### 3.5. FTIR and the Secondary Structure

**Figure 5:**
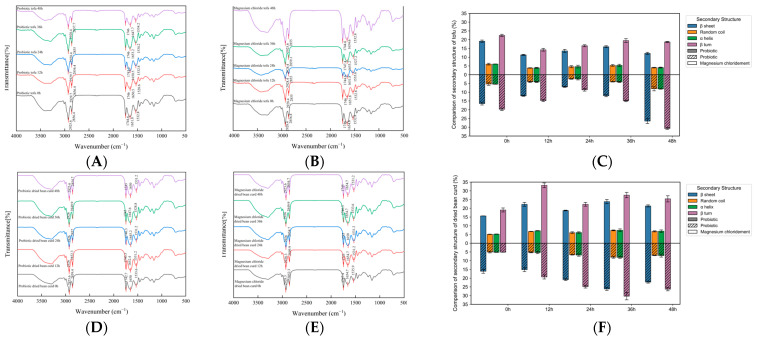
*FTIR* (**A**) Probiotic tofu. (**B**) Magnesium chloride tofu. (**C**) Comparison of secondary structure of two kinds of tofu samples over time. (**D**) Probiotic dried bean curd. (**E**) Magnesium chloride dried bean curd. (**F**) Comparison of secondary structure of two kinds of dried bean curd samples over time.

Fourier transform infrared spectroscopy (FTIR) has a high sensitivity, resolution, and signal-to-noise ratio and accurate frequency measurement capabilities. It can analyze protein secondary structure changes based on the interaction between infrared radiation and molecular vibration/rotation. This study investigated probiotic treatment effects on soymilk protein structure using amide I band C=O stretching vibration (1600–1700 cm^−1^). Figure 5 demonstrated significantly higher absorption intensity in the treatment group within this band, indicating significant alterations in soybean protein’s peptide bond carbonyl vibration modes. Quantitative analysis of the *β*-sheet, random coil, *α*-helix, and *β*-turn bands revealed dynamic secondary structure content changes. The spectral characteristics were determined by protein peptide chain hydrogen bonding properties, with vibration frequencies directly correlating to specific secondary structures (*α*-helix at 1650–1660 cm^−1^).

Figure 5C demonstrates that amide I protein structures in the tofu primarily consisted of *β*-sheets and *β*-turns. During storage, both tofu types exhibited initial decreases followed by increases in these structures, while random coil and *α*-helix contents remained stable around 5%. Specifically, *β*-turns in the magnesium chloride tofu increased from 19.75% to 30.70% (0–48 h), contrasting with the probiotic tofu’s *β*-turns’ decrease from 22.41% to 18.68% (0–48 h). Infrared spectra revealed irregular peak fluctuations in both samples, potentially attributable to hydrogen bond disruption by L-lactic acid in acid slurry coagulant. This structural unfolding led to *α*-helix/random coil transformations, with *β*-sheets increasing from 16.51% to 26.49%, indicating enhanced protein secondary structure order through probiotic-fermented yellow slurry water coagulation. The attenuated *β*-sheet/*α*-helix ratios observed in the FTIR spectra of the probiotic tofu align with its electrophoretic stability, suggesting that lactic acid fermentation preferentially disrupts non-covalent interactions without altering covalent subunit integrity, contrasting with the magnesium chloride tofu’s progressive aggregation linked to microbial *β*-sheet formation [29].

Figure 5F reveals *β*-sheets and *β*-turns as dominant protein structures in amide I-containing dried bean curd (DBC). Both types exhibited an initial increase followed by a decrease to equilibrium in these structures during storage, while random coil and *α*-helix structures maintained ~7% stability. During storage, *β*-turns in the probiotic DBC increased from 19.03% to 25.40% (0–48 h), while the magnesium chloride DBC showed a more pronounced increase from 5.14% to 26.31% (0–48 h). Notably, the probiotic DBC initially exhibited 0.45% lower *β*-sheets but 13.89% higher *β*-turns than the magnesium chloride DBC. This structural shift is likely attributable to L-lactic acid-induced hydrogen bond disruption in the acid slurry coagulant, promoting the expansion of other structures into *β*-turns. The data demonstrate that probiotic-fermented yellow slurry water coagulation enhances protein secondary structure order in DBC, with *β*-turns forming the primary components connected through secondary bonds to establish stable gel networks. The observed increase in *β*-turn content in the probiotic DBC aligns with previous findings where nano-sized dietary fiber incorporation promoted *α*-helix and *β*-turn stabilization, suggesting a synergistic effect between lactic acid-induced structural rearrangement and fiber-mediated molecular interactions on protein secondary ordering [30].

### 3.6. Effect of Probiotics Fermented Yellow Slurry Water on Microstructure

**Figure 6:**
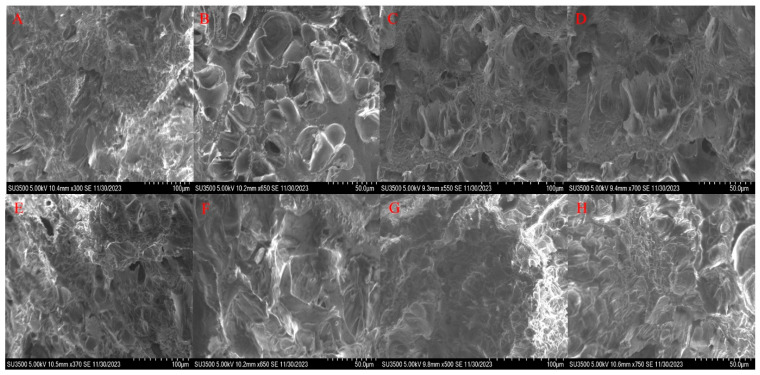
The SEM images of different tofu and dried bean curd samples. (**A**) Magnesium chloride tofu × 300. (**B**) Probiotic tofu × 650. (**C**) Magnesium chloride tofu × 550. (**D**) Probiotic tofu × 700. (**E**) Magnesium chloride dried bean curd × 370. (**F**) Probiotic dried bean curd × 650. (**G**) Magnesium chloride dried bean curd × 500. (**H**) Probiotic dried bean curd × 750.

In order to explore the effect of probiotic acid slurry and magnesium chloride coagulant on the microstructure of tofu, it was found that the two kinds of tofu were porous structures by SEM, but there were significant differences. It can be seen from Figure 6A,C that the gel structure of the magnesium chloride-spotted tofu was relatively continuous and evenly distributed. The magnesium chloride tofu promoted protein aggregation into larger particles due to magnesium ions, resulting in larger pores between protein aggregates. The gel structure was continuous but the distribution uniformity was poor. The observed larger pores in magnesium chloride-coagulated tofu compared to the denser network of GYP tofu in one study could be attributed to distinct aggregation patterns induced by divalent cations versus calcium ions, where magnesium’s lower ionic strength might facilitate looser protein clustering during coagulation [31]. Acid slurry coagulant (containing metabolites of *Saccharomyces cerevisiae* and *Lacticaseibacillus rhamnosus*) can promote more uniform protein distribution, form a more continuous and dense gel network, and have smaller pores. This is due to the fact that H^+^ in the acid pulp neutralizes the surface charge of the protein and forms a tight three-dimensional structure through hydrophobic interactions and disulfide bonds. In addition, the acid slurry coagulant can reduce the risk of processing pollution and improve the edible and nutritional value of tofu.

SEM analysis revealed distinct microstructural differences between DBC coagulated with probiotic acid slurry and magnesium chloride, as shown in Figure 6. Both DBC types exhibited porous structures, though the magnesium chloride samples (Figure 6F,H) displayed larger pores with continuous uniform gel networks, likely due to electrostatic interaction-induced rapid protein aggregation forming loose matrices. In contrast, the acid slurry DBC, as shown in Figure 6E,G, showed smaller, denser pores with orderly protein alignment and refined 3D networks, attributed to enhanced protein cross-linking by microbial metabolites in the acid slurry. While magnesium chloride coagulation typically yielded higher gel hardness, pore characteristics were concentration-dependent. Notably, acid slurry coagulant demonstrated dual advantages: reduced processing contamination risks and enhanced nutritional and textural stability in DBC. These microstructural variances establish a scientific basis for coagulant optimization in plant-protein gels.

## 4. Conclusions

This study demonstrates that probiotic fermentation utilizing soybean whey (an industrial by-product) significantly enhances the nutritional and functional properties of soybean products through multidimensional structural modulation. The synergistic action of *Lacticaseibacillus rhamnosus* and *Saccharomyces cerevisiae* in probiotic soymilk not only improved volatile aroma profiles via enzymatic protein degradation into bioactive peptides (SDS-PAGE confirmed) but also exemplifies by-product valorization by transforming agro-industrial waste into value-added functional foods. This bioconversion strategy contributes to circular food systems by minimizing waste and optimizing resource efficiency, while aligning with sustainable dietary innovations through microbial-driven nutrient enhancement. Probiotic fermentation mitigates bean product discoloration through the stabilization of microbial–pigment interactions, exhibiting 23% reduced ΔE variation and diminished brightness loss. Chromatic–proteolytic correlations substantiate microbial metabolism as the predominant reason underlying quality deterioration in soybean protein matrices. FTIR analysis revealed that probiotic processing reshaped soybean protein’s secondary structure equilibrium, as evidenced by dynamic shifts in the *β*-sheet/*α*-helix ratios and C=O vibration mode alterations. This structural reorganization provides a spectroscopic basis for optimizing fermentation-induced protein modifications. Probiotic-coagulated tofu exhibited superior microstructure, water retention, and protein ordering during 48 h storage, with residual microbial metabolites contributing to elevated protein content versus that in magnesium chloride controls and *β*-turn conformation progressively increasing as the dominant secondary structure. Crucially, coagulant type did not alter protein subunit stability, confirming the universal applicability of probiotic-fermented yellow slurry water. These findings establish a scientific foundation for developing probiotic-enhanced soybean products with improved nutritional value and storage characteristics.

## Figures and Tables

**Table 1:** Determination of the number of viable bacteria in fermented yellow slurry water for 24 h.

Sample	*Lacticaseibacillus rhamnosus* (CFU/mL)	*Saccharomyces cerevisiae* (CFU/mL)	Total Viable Counts (CFU/mL)
AS1	2.37 × 10^11^	1.64 × 10^11^	4.01 × 10^11^
AS2	5.30 × 10^8^	8.60 × 10^8^	1.39 × 10^9^
AS3	1.63 × 10^11^	2.59 × 10^11^	4.22 × 10^11^
AS4	2.83 × 10^11^	5.07 × 10^12^	5.35 × 10^12^
AS5	1.37 × 10^11^	6.27 × 10^10^	2.00 × 10^11^

## Data Availability

The original contributions presented in the study are included in the article, further inquiries can be directed to the corresponding author.
